# Supplementary material for: Pragmatic, quasi-experimental, pseudo-randomized clinical trial to assess the impact of patient safety monitors on clinical and patient safety outcomes: The Akershus Clinical Trial (ACT) 1
Source: PLoS One. 2025 Oct 22;20(10):e0335052. doi: 10.1371/journal.pone.0335052 (PMC12543108; doi:10.1371/journal.pone.0335052)
Supplement: S1 Table — *p-value for difference between intervention and control group, t-test for age, Chi-square test otherwise. (DOCX) [file pone.0335052.s001.docx]

**S1 Table: Baseline characteristics for bays where patient safety monitors were not implemented**

|  | Intervention period (n=3214) | Control period (n=2947) | p-value* |
| --- | --- | --- | --- |
| Age, years | 65 [48-78] | 64 [49-76] | 0.10 |
| Female sex, n (%) | 2470 (83.8) | 2260 (76.7) | 0.90 |
| Body mass index, kg/m2 |  |  |  |
| Charlson score, n (%) |  |  | 0.25 |
| *0* | 1893 (64.2) | 1802 (61.1) | 0.07 |
| *1* | 498 (15.5) | 415 (12.9) | 0.12 |
| *2* | 352 (11.9) | 318 (10.8) | 0.84 |
| *3* | 159 (4.9) | 160 (5.0) | 0.39 |
| *≥4* | 312 (9.7) | 252 (7.8) | 0.12 |
| Emergency hospitalization, n (%) | 2404 (74.8) | 2065 (64.3) | <0.01 |
| Medical history, n (%) |  |  |  |
| *Cerebrovascular disease* | 351 (10.9) | 281 (8.7) | 0.08 |
| *Heart failure* | 195 (6.1) | 162 (5.0) | 0.37 |
| *Myocardial infarction* | 213 (6.6) | 203 (6.3) | 0.72 |
| *Diabetes mellitus* | 266 (8.3) | 237 (7.4) | 0.78 |
| *Chronic pulmonary disease* | 327 (10.2) | 279 (8.7) | 0.38 |
| *Malignancy* | 384 (11.9) | 312 (9.7) | 0.10 |
| *Dementia* | 89 (2.8) | 82 (2.6) | 1.00 |
| *Renal disease* | 121 (3.8) | 126 (3.9) | 0.34 |
| *Peripheral vascular disease* | 179 (5.6) | 171 (5.3) | 0.73 |

**p-value for difference between intervention and control group, t-test for age, Chi-square test otherwise.*
