# Supplementary material for: Pragmatic, quasi-experimental, pseudo-randomized clinical trial to assess the impact of patient safety monitors on clinical and patient safety outcomes: The Akershus Clinical Trial (ACT) 1
Source: PLoS One. 2025 Oct 22;20(10):e0335052. doi: 10.1371/journal.pone.0335052 (PMC12543108; doi:10.1371/journal.pone.0335052)
Supplement: S2 Table — All analyses adjusted for age, sex, Charlson comorbidity score, cause of index admission, and admission period (2019–2020 vs. 2021–2022). (DOCX) [file pone.0335052.s002.docx]

**S2 Table: Odds ratio (OR) for safety endpoint in the period after implementation of patient safety monitors (PSM) compared to before for intervention wards and control wards (where PSM was not implemented)**

|  | **Intervention wards** | | | **Control wards** | | |  |
| --- | --- | --- | --- | --- | --- | --- | --- |
|  | **OR** | **95% CI** | **p** | **OR** | **95% CI** | **p** | **p-for-interaction** |
| Nutritional screening within 24 hours | 0.67 | 0.53-0.83 | <0.001 | 0.76 | 0.64-0.90 | 0.002 | 0.402 |
| Nutritional screening within initial hospital stay | 0.66 | 0.55-0.79 | <0.001 | 0.66 | 0.57-0.77 | <0.001 | 0.984 |
| Fall screening within 24 hours | 0.64 | 0.52-0.78 | 0.001 | 0.77 | 0.65-0.90 | 0.002 | 0.200 |
| NEWS score within 24 hours | 1.08 | 0.80-1.44 | 0.622 | 1.23 | 0.97-1.57 | 0.092 | 0.486 |
| Dalteparin during hospital stay | 0.90 | 0.80-1.01 | 0.072 | 1.02 | 0.92-1.13 | 0.727 | 0.181 |
| Medication reconciliation | 16.1 | 9.87-28.5 | <0.001 | 14.3 | 7.18-34.0 | <0.001 | 0.760 |

All analyses adjusted for age, sex, Charlson comorbidity score, cause of index admission, and admission period (2019–2020 vs. 2021–2022).
