# Supplementary material for: Pragmatic, quasi-experimental, pseudo-randomized clinical trial to assess the impact of patient safety monitors on clinical and patient safety outcomes: The Akershus Clinical Trial (ACT) 1
Source: PLoS One. 2025 Oct 22;20(10):e0335052. doi: 10.1371/journal.pone.0335052 (PMC12543108; doi:10.1371/journal.pone.0335052)
Supplement: S3 Table — All analyses adjusted for age, sex, Charlson comorbidity score, cause of index admission, and admission period (2019–2020 vs. 2021–2022). (DOCX) [file pone.0335052.s003.docx]

**S3 Table: Number of NEWS scores taken during the index hospital stay for the intervention wards and control wards (where PSM was not implemented)**

|  | **Median (IQR) 2019-2020** | **Median (IQR) 2021-2022** | **IRR** | **95% CI** | **p-value** |
| --- | --- | --- | --- | --- | --- |
| Intervention wards in intervention period | 10 (5-18) | 11 (5-20) | 1.01 | 0.95-1.07 | 0.809 |
| 2021+2022 vs. 2019+2020 (intervention wards) | 12 (5-20) | 12 (5-22) | 1.08 | 1.03-1.13 | 0.001 |
| 2021+2022 vs. 2019+2020 (control wards) | 9 (4-17) | 10 (5-18) | 1.08 | 1.03-1.13 | 0.001 |

All analyses adjusted for age, sex, Charlson comorbidity score, cause of index admission, and admission period (2019–2020 vs. 2021–2022).
