# Supplementary material for: Pragmatic, quasi-experimental, pseudo-randomized clinical trial to assess the impact of patient safety monitors on clinical and patient safety outcomes: The Akershus Clinical Trial (ACT) 1
Source: PLoS One. 2025 Oct 22;20(10):e0335052. doi: 10.1371/journal.pone.0335052 (PMC12543108; doi:10.1371/journal.pone.0335052)
Supplement: S4 Table — All analyses adjusted for age, sex, Charlson comorbidity score, cause of index admission, and admission period (2019–2020 vs. 2021–2022). (DOCX) [file pone.0335052.s004.docx]

**S4 Table: Time-to-event analyses**

|  | **With interaction** | | | **Intervention wards** | | | | **Control wards** | | | |
| --- | --- | --- | --- | --- | --- | --- | --- | --- | --- | --- | --- |
|  | **HR** | **95% CI** | **p** | **HR** | **95% CI** | **p** | **HR** | | **95% CI** | **p** |  |
| 30-day mortality | 0.90 | 0.53-1.53 | 0.685 | 0.90 | 0.65-1.24 | 0.517 | 1.00 | | 0.65-1.54 | 0.989 |  |
| 30-day readmission | 1.15 | 0.88-1.52 | 0.309 | 1.14 | 0.93-1.40 | 0.204 | 0.99 | | 0.82-1.19 | 0.901 |  |
| 1-year mortality | 0.97 | 0.75-1.25 | 0.810 | 0.97 | 0.83-1.14 | 0.747 | 1.00 | | 0.82-1.22 | 0.981 |  |
| 1-year readmission | 0.98 | 0.80-1.20 | 0.844 | 0.92 | 0.80-1.07 | 0.270 | 0.94 | | 0.82-1.07 | 0.352 |  |

All analyses adjusted for age, sex, Charlson comorbidity score, cause of index admission, and admission period (2019–2020 vs. 2021–2022).
