# Supplementary material for: Pragmatic, quasi-experimental, pseudo-randomized clinical trial to assess the impact of patient safety monitors on clinical and patient safety outcomes: The Akershus Clinical Trial (ACT) 1
Source: PLoS One. 2025 Oct 22;20(10):e0335052. doi: 10.1371/journal.pone.0335052 (PMC12543108; doi:10.1371/journal.pone.0335052)
Supplement: S1 Appendix — (PDF) [file pone.0335052.s005.pdf]

Monitoring of Data Extraction

Project Title: AKERSHUS CLINICAL TRIAL (ACT) 1: RETROSPECTIVE STUDY TO ASSESS WHETHER PATIENT SAFETY MONITORS THAT PRESENT REAL-TIME ELECTRONIC HEALTH DATA CAN IMPROVE CLINICAL AND PATIENT SAFETY OUTCOMES

Project Leader: Professor Helge Røsjø

Responsible Data Manager: Olav Kyrre Lenvik

<u>Before Conducting the Extraction:</u>

**Monitoring Tasks**	**Performed by**	**Signature**	**Date**
The need for assistance from researchers in ACR has been assessed. ACR has been contacted via the coordinator if necessary.	Not applicable		
It has been validated that the necessary approvals are in place from:			
The Directorate of Health for exemption from confidentiality	Helge Røsjø, Magnus N Lyngbakken	Helge Røsjø<br>Magnus N. Lyngbakken	26.05.2023
REK	Helge Røsjø, Magnus N Lyngbakken	Helge Røsjø<br>Magnus N. Lyngbakken	08.02.2023
Data Protection Officer, Akershus University Hospital	Helge Røsjø, Magnus N Lyngbakken	Helge Røsjø<br>Magnus N. Lyngbakken	05.06.2023
A meeting has been held with the clinician to establish a common understanding of the desired data and what the data manager is able to extract.	Kristian Berge, Torbjørn Wisløff	Kristian Berge	10.08.2023

After Data Extraction is Completed and Before Delivery:

Monitoring Tasks	Performed by	Signature	Date
A sanity test has been conducted based on the clinician's expectations regarding the number of patients, etc.	Kristian Berge,<br>Rune Bruhn Jakobsen	Kristian Berge<br>[illegible]	15.05.2024
A data manager from the Department of Analytics, who has not been involved in the project, has performed a code review to identify potential errors in the script.	Haldor Husby	[illegible]	08.04.2024
The data manager has reviewed 20 random samples.	Kristine Lippestad	Kristine Lippestad	09.04.2024
Various time periods have been reviewed to detect possible changes in registration practice.<br><br>The following periods have been checked: 2019 - 2022	Kristine Lippestad	Kristine Lippestad	09.04.2024
A clinician has conducted 10 random samples, where values were manually verified in the data system (DIPS, MV, etc.) and compared with the values provided in the data set. The number of random samples was adjusted based on the extraction size.	Kristian Berge	Kristian Berge	10.05.2024
A meeting has been held with the data manager, clinician, and statistician to review all variables and establish a shared understanding of what the values represent.	Olav Kyrre Lenvik,<br>Ane Stenset,<br>Johanna Austeen Gjestland,<br>Torbjørn Wisløff,<br>Kristine Lippestad	Kristine Lippestad	19.10.2023
The occurrence of missing data has been assessed. If necessary, the statistician has conducted a pilot study and verified that there is sufficient data to answer the endpoints.	Torbjørn Wisløff	[illegible]	08.12.2023
Scripts and a copy of the data have been stored in a secure location by the data manager to maintain a copy of the data at time of delivery.	Olav Kyrre Lenvik	[illegible]	25.10.2023
The dataset has been delivered to: TSD	Olav Kyrre Lenvik	[illegible]	25.10.2023
