# Supplementary material for: Pragmatic, quasi-experimental, pseudo-randomized clinical trial to assess the impact of patient safety monitors on clinical and patient safety outcomes: The Akershus Clinical Trial (ACT) 1
Source: PLoS One. 2025 Oct 22;20(10):e0335052. doi: 10.1371/journal.pone.0335052 (PMC12543108; doi:10.1371/journal.pone.0335052)
Supplement: S2 Appendix — (PDF) [file pone.0335052.s006.pdf]

Administrative information:

Sponsor name	Akershus University Hospital, Norway
Sponsor address	Sykehusveien 25<br>1478 Lørenskog, Norway
EudraCT number / REC no	[]
Trial title	Akershus Clinical Trial (ACT) 1: Retrospective study to assess whether automatic safety monitors that present real-time electronic health data can improve clinical and patient safety outcomes
Trial ID	[]
Trial registration number	[]

SAP and protocol version:

SAP version and date:	[This SAP is version 1.0, dated 11 January 2023]
Protocol version	[This document has been written based on information contained in the study protocol version 2, dated 11th January 2023]

SAP revision history:

Protocol version	SAP version	Section number changed	Description and reason for change	Date changed
[1.2]	[1.0]	[NA]	[NA]	[11.01.2023]

SIGNATURE PAGE

PRINCIPAL/COORDINATING INVESTIGATOR:

Inge Skråmm, MD, PhD
Division of Orthopedic Surgery

Signature

Date (dd/mmm/yyyy)

TRIAL STATISTICIAN:

Torbjørn Wisløff, MSc, PhD
Affiliation

Signature

Date (dd/mmm/yyyy)

ABBREVIATIONS

AIC	Akaikes Information Criterion
CCU	Cardiac Care Unit
EHR	Electronic Health Record
GLM	Generalized linear model
ICU	Intensive Care Unit
IMCU	Intermediate Care Unit
NEWS2	National Early Warning Score
PCU	Postoperative Care Unit
PSM	Patient Safety Monitor
SD	Standard deviation
TP	Time point
TSD	Services for sensitive data, University of Oslo

TABLE OF CONTENTS

1 INTRODUCTION ... 5
1.1 Background and Rationale ... 5
1.2 Intervention(s) ... 5
1.3 Trial Objectives ... 5
2 TRIAL METHODS ... 6
2.1 Trial Design ... 6
2.2 Randomisation ... 6
2.3 Statistical Framework ... 7
2.4 Timing of Outcome Assessments ... 7
2.5 Statistical Interim Analyses and Stopping Guidance ... 7
2.6 Timing of Main Analysis ... 7
3 TRIAL POPULATION ... 8
3.1 Screening Data, Eligibility and Recruitment ... 8
3.2 Baseline Patient Characteristics ... 8
3.3 Withdrawal/Follow-up ... 8
3.4 Adherence and Protocol Deviations ... 8
3.5 Analysis Populations ... 8
4 OUTCOME DEFINITIONS ... 9
4.1 Outcome definitions ... 9
4.2 Overview of Outcomes ... 9
5 ANALYSIS METHODS ... 10
5.1 Methods for Primary Outcome ... 10
5.2 Methods for Dichotomous Secondary Outcomes ... 11
5.3 Methods for Time to Event Secondary Outcomes ... 11
5.4 Methods for Secondary Outcomes with count data ... 11
5.5 Methods for Cost data ... 12
5.6 Sample size ... 12
6 STATISTICAL SOFTWARE ... 12
7 REFERENCES ... 12
7.1 Literature References ... 12

1 Introduction

1.1 Background and Rationale

Patient injuries and adverse events are recognized as main challenges in the Norwegian National Action Plan for Patient Safety and Quality Improvement. The Norwegian Health Directory´s patient safety program "In safe hands 24-7" recommends huddle board as a tool to monitor patient safety measures, as defined in the program. However, there is limited information in the literature whether huddle boards impact clinical and patient safety outcomes. In addition, electronic health records now provide opportunity to integrate data for individual patients and report these back in structured format to clinical personnel on large screens, so-called Patient Safety Monitors (PSMs). Hence, we hypothesized that the use of PSMs in hospital wards in a Norwegian Orthopedic Department will reduce hospital length of stay and improve clinical and patient safety outcomes.

1.2 Intervention(s)

Intervention: PSM implemented at two orthopedic wards

Control: Standard of care in orthopedic wards without PSM

1.3 Trial Objectives

1.3.1 Primary Objective

To determine whether the use of PSM will reduce hospital length of stay (hours) compared to historical control patients

1.3.2 Secondary Objectives

The secondary objectives of this study are:

- To determine whether the use of PSM will reduce 30-day re-admission rate for any cause compared to historical control patients
- To determine whether the use of PSM will reduce 30-day all-cause mortality compared to historical control patients
- To determine whether the use of PSM will reduce 1-year re-admission rate for any cause compared to historical control patients
- To determine whether the use of PSM will reduce 1-year all-cause mortality compared to historical control patients
- To determine whether the use of PSM will reduce the number of ICU/CCU admissions during the index hospitalization compared to historical control patients

- To determine whether the use of PSM will increase nutritional screening within 24 h and documented in the Electronic Health Record (EHR) after admission compared to historical control patients
- To determine whether the use of PSM will increase the occurrence of nutritional support measures in the treatment plan documented in the EHR compared to historical control patients
- To determine whether the use of PSM will increase fall screening documented in the EHR within 24 h after admission compared to historical control patients
- To determine whether the use of PSM increases the number of patients subjected to National Early Warning Score (NEWS) 2 assessment documented in the EHR compared to historical control patients
- To determine whether the use of PSM increases the total number of NEWS 2 assessments documented in the EHR during in patients the index hospitalization compared to historical control patients
- To determine whether the use of PSM increases the prevalence of anti-coagulation therapy compared to historical control patients
- To determine whether the use of PSM increases the prevalence of patients receiving anti-coagulation therapy at discharge from the index hospitalization compared to historical control
- To estimate total resource use during hospitalization for intervention and control patients
- To estimate 30-day cost for intervention and control patients, including current admission and readmissions
- To determine whether the use of PSM increases the prevalence of medication reconciliation compared to historical control patients.

2 Trial Methods

2.1 Trial Design

Single-center, pragmatic, pseudo-randomized controlled trial with in silico-control group for a non-pharmacological intervention.

2.2 Randomisation

The Division of Orthopedic Surgery at Ahus consists of two 7-days wards, S105 and S205, with four areas each: area A, B, C and D. We implemented PSM at area A and B at ward S105 and S205 in 2021 (intervention group). To obtain a control group with similar patient composition, we will compare the results for the intervention group with an in silico-control group, which is patients admitted to area A and B at ward S105 and S205 in 2020 (prior to implementation of PSM).

2.3 Statistical Framework

2.3.1 Hypothesis Test

This trial is designed to estimate the difference in hospital length of stay in areas with PSMs compared to areas without PSMs.

2.3.2 Confidence Intervals and p-values

All efficacy estimates will be presented with two-sided 95% confidence intervals. The primary outcome will be presented with confidence interval and two-sided p-value. As there is only one primary objective in this trial, there will be no adjustments for multiplicity, and no p-values reported for other than the primary outcome.

2.4 Timing of Outcome Assessments

For analysis and tabulation purposes, we define study time points as

**Time Point Label**	**Definition (Day window)**
TP1. Baseline	Information on admission
TP2. Discharge	Hospital stay
TP3. 30 days	TP2 + 30 days
TP4. 1 year	TP2 + 365 days<br>• For patients admitted between Mar 1st 2019 and Feb 28th 2020: (date – 1) one year after admission<br>• All other patients: same date one year after admission

If more than one visit fall into the same time point interval, information on all visits will be used in the analyses.

2.5 Statistical Interim Analyses and Stopping Guidance

There will be no interim analyses in this trial.

2.6 Timing of Main Analysis

Data for the primary objective can be extracted at the earliest 60 days after last patient is recruited, i.e. March 1st 2023.

3 Trial Population

3.1 Screening Data, Eligibility and Recruitment

All patients hospitalized at the Orthopedic Center, from Jan 1, 2019, to Dec 31, 2022 will be included.

3.2 Baseline Patient Characteristics

The patient demographics and baseline characteristics to be summarised separately for treatment and control groups include age, sex, comorbidities (atrial fibrillation, stroke, hypertension, coronary artery disease, heart failure, myocardial infarction, diabetes mellitus, chronic obstructive pulmonary disease, anaemia, cancer, dementia, obstructive sleep apnoea, chronic kidney failure, peripheral artery disease), Charlson Comorbidity Index, and cause of index admission. Categorical data will be presented as absolute frequencies and percentages. For continuous data, N, mean±SD or median (25th and 75th percentiles).

3.3 Withdrawal/Follow-up

The study is regarded as quality improvement and no consent is given, which implies withdrawal of consent will not be possible.

Outcomes with long-term follow-up (1 year) will be assessed based on data in the EHR. We will not be able to know whether patients have moved, or have been treated at other hospitals. Patients who in practice are losses to follow-up will therefore be assumed as no events has occurred.

Time to event variables and Kaplan-Meier product-limit estimates will be presented stratified by intervention group. Risk of events in time-to-event models will be assessed by adjusted Cox regression. The analyses will be conducted on all patient data at the time of inclusion of the last patient.

3.4 Adherence and Protocol Deviations

3.4.1 Adherence to Allocated Treatment

Patients will be analysed based on the area they were admitted.

3.4.2 Protocol Deviations

Not applicable. If patients are moved between units in the hospital, they will be analysed based on their initial admission.

3.5 Analysis Populations

All the following conditions must apply to the prospective patient at screening prior to inclusion in the study:

- Age ≥18 y
- Admitted to the designated areas during the study period

Patients will be excluded from the study if they meet any of the following criteria:

- Previously included into the study (in case of patients presenting with a second hospitalization during the study period)

4 Outcome Definitions

4.1 Outcome definitions

4.1.1 Hospital length of stay

Time from admission to discharge as reported in the EHR.

4.1.2 Readmission

Patient admitted to hospital, i.e. not outpatient

4.1.3 All cause mortality

Patient reported as dead

4.1.4 ICU/ICMU/PCU/CCU admission

Patient registered in ICU/ICMU/PCU/CCU

4.1.5 Nutritional screening

Patient exposed to nutritional screening and documented in the Electronic Health Record (EHR)

4.1.6 Fall screening

Patient exposed to fall screening and documented in the Electronic Health Record (EHR)

4.1.7 National early warning 2 assessment

Patient subjected to National Early Warning Score (NEWS) 2 assessment documented in the EHR

4.1.8 In-hospital anti-coagulation therapy

Patient given dalteparin in hospital

4.1.9 Total resource use during hospitalisation

Total resource use as reported in the EHR based on the costs-per-patient system

4.1.10 30-day cost

Adding to 4.1.9 all costs of other contacts with AHUS during 30 days follow-up

4.2 Overview of Outcomes

Level	Outcome	Timeframe	Type
Primary	Length of stay	Index hospitalisation	Continuous
Secondary	Time to re-admission	30 days & 1 year	Time to event
	Time to death	30 days & 1 year	Time to event
	ICU/ICMU/PCU/CCU admissions	Index hospitalisation	Count
	Nutritional screening	24 hours	Dichotomous
	Nutritional support measures in the treatment plan	Index hospitalisation	Dichotomous
	Fall screening	24 hours	Dichotomous
	NEWS 2 assessment	Index hospitalisation	Dichotomous
	NEWS 2 assessment	Index hospitalisation	Count
	In-hospital anti-coagulation therapy	Index hospitalisation	Dichotomous
	In-hospital anti-coagulation therapy	At discharge	Dichotomous
	Hospital resource use	Index hospitalisation & 30 days	Costs (NOK)
	Medication reconciliation	Index hospitalisation	Dichotomous

5 Analysis Methods

5.1 Methods for Primary Outcome

5.1.1 Descriptive Statistics

Descriptive statistics will include number and percentage by treatment group

5.1.2 Primary Inferential Analysis

Hospital length of stay for patients in areas with PSM will compared to historical control patients from the same areas using generalised linear regression with gamma family and log link adjusting for age, sex, Charlson Comorbidity Index, and cause of index admission.

5.1.3 Effect Estimates

The primary effect estimate will be the difference between intervention and control, presented with a two-sided 95% confidence interval and p-value.

5.1.4 Assumption Checks and Alternative Analyses

For the primary outcome, we will check the assumption of normality of residuals. If GLM using Gaussian family and identity link function provides lower AIC and a QQ plot without any clear skewness, this will be the primary analysis.

5.1.5 Missing Data

For most outcomes, missing data is not possible, as these are either dichotomous or count data, where no value will be interpreted as zero. For the primary outcome, missing data occur if there is no discharge date for a patient. These patients will be excluded in the primary analysis. Following the logic of worst case imputation for the primary outcome, a separate analysis will be performed where patients without discharge date will be given the maximum number of hospital days among the population.

5.1.6 Sensitivity Analyses

Some sensitivity analyses will be performed, including analyses with different handling of missing data.

5.1.7 Subgroup Analyses

No predefined subgroup analyses are planned. Potential effects of subgroups may be handled through interaction effects in regression analyses.

5.2 Methods for Dichotomous Secondary Outcomes

All dichotomous outcomes will be analysed with logistic regression adjusting for age, sex, comorbidities, and cause of index admission.

5.3 Methods for Time to Event Secondary Outcomes

All time to event outcomes will be analysed with Cox regression adjusting for age, sex, comorbidities, and cause of index admission. Patients still in hospital at the end of follow-up will be censored at maximum follow-up (30 days and 1 year). Time-to-event outcomes will also be presented with a Kaplan-Meier curve with a separate curve for each intervention.

5.4 Methods for Secondary Outcomes with count data

All count data will be analysed with Poisson regression adjusting for age, sex, Charlson Comorbidity Index, and cause of index admission.

5.5 Methods for Cost data

All cost data will be analysed with generalised linear regression with gamma family and log link adjusting for age, sex, Charlson Comorbidity Index, and cause of index admission.

5.6 Sample size

Sample size calculations are not applicable for this study as we use retrospective design. However, we have calculated power of the data to demonstrate a potential change on the primary endpoint. Data from the Norwegian Patient Registry indicates that the mean length of stay in orthopedic wards is 4 days (standard deviation 2.5 days). We define a clinically relevant reduction in hospital length of stay to be at least 1 day (24 hours). Based on these numbers and expecting 1:1 ratio between hospitalized patients during the control period (2019-2020) and implementation period (2021-2022), we will need a minimum of 100 patients in the intervention arm and 100 patients in the in silico-control arm to have >80% probability to detect a difference with significance level 0.05. We know that >500 unique patients were hospitalized in the designated areas related to the study population, hence, we should have sufficient statistical power to detect a possible difference on the primary endpoint after implementation of PSM.

6 Statistical Software

All statistical analyses will be performed in R using Rstudio with the version provided by TSD.

7 References

7.1 Literature References

1. Guidelines for the Content of Statistical Analysis Plans in Clinical Trials. 2017;318(23):2337-2343. doi:10.1001/jama.2017.18556.
