# Supplementary material for: Pragmatic, quasi-experimental, pseudo-randomized clinical trial to assess the impact of patient safety monitors on clinical and patient safety outcomes: The Akershus Clinical Trial (ACT) 1
Source: PLoS One. 2025 Oct 22;20(10):e0335052. doi: 10.1371/journal.pone.0335052 (PMC12543108; doi:10.1371/journal.pone.0335052)
Supplement: S3 Appendix — (PDF) [file pone.0335052.s007.pdf]

AKERSHUS CLINICAL TRIAL (ACT) 1: RETROSPECTIVE STUDY TO ASSESS WHETHER PATIENT SAFETY MONITORS THAT PRESENT REAL-TIME ELECTRONIC HEALTH DATA CAN IMPROVE CLINICAL AND PATIENT SAFETY OUTCOMES

Protocol Identification Number: xxx

EudraCT Number: n.a.

ClinicalTrials.gov Identifier: xx

SPONSOR: **Akershus University Hospital**
1478 Lørenskog, Norway
Tel : +47 679 60 000

PRINCIPAL INVESTIGATOR: **Inge Skråmm**
MD, PhD
Division of Orthopedic Surgery
Akershus University Hospital
Sykehusveien 25
1478 Lørenskog, Norway
Tel: +47 922 12 722
E-mail: inge.skramm@ahus.no

PROTOCOL VERSION NO. 1
Version #2

CONTACT DETAILS

**Sponsor:**	**Akershus University Hospital**<br>Division of Orthopedic Surgery<br>1478 Lørenskog, Norway<br>Tel: +47 679 60 000
**Principal Investigator:**	**Inge Skråmm**<br>MD, PhD<br>Division of Orthopedic Surgery<br>Akershus University Hospital<br>Sykehusveien 25<br>1478 Lørenskog, Norway<br>Tel: +47 922 12 722<br>E-mail: inge.skramm@ahus.no
**Co-Principal Investigator**	**Professor Helge Røsjø**<br>MD, PhD<br>Division of Research and Innovation<br>Akershus University Hospital<br>Sykehusveien 25<br>1478 Lørenskog, Norway<br>Tel: +47 915 45 864<br>E-mail: helge.rosjo@medisin.uio.no
**Project coordinator**	**Johanna A. Gjestland**<br>MSc<br>Division of Orthopedic Surgery<br>Akershus University Hospital<br>Sykehusveien 25<br>1478 Lørenskog, Norway<br>Tel: +47 908 21 840<br>E-mail: johanna.gjestland@ahus.no
**Investigator:**	**Associate Professor Magnus Lyngbakken**<br>MD, PhD<br>Division of Medicine<br>Akershus University Hospital<br>Sykehusveien 25<br>1478 Lørenskog, Norway<br>Tel: +47 93 40 88 37<br>E-mail: magnus.lyngbakken@medisin.uio.no

Investigator: **Kristian Berge**
MD
Division of Medicine
Akershus University Hospital
Sykehusveien 25
1478 Lørenskog, Norway
Tel: +47 412 83 128
E-mail: kristian.berge@gmail.com

Investigator: **Anett H Ottesen**
PhD
Division of Research and Innovation
Akershus University Hospital
Sykehusveien 25
1478 Lørenskog, Norway
Tel: +47 984 52 582
E-mail: a.h.ottesen@medisin.uio.no

Investigator: **Olav Lenvik**
BSc
Department of Analytics, Division of Economy and Finance
Akershus University Hospital
Sykehusveien 25
1478 Lørenskog, Norway
Tel: +47 974 96 113
E-mail: olav.lenvik@ahus.no

Investigator: **Lars Åge Møgster**
MSc
Department of Analytics, Division of Economy and Finance
Akershus University Hospital
Sykehusveien 25
1478 Lørenskog, Norway
Tel: +47 991 51 874
E-mail: lars.age.mogster@ahus.no

Investigator: **Torbjørn Wisløff**
MSc, PhD
Division of Research and Innovation
Akershus University Hospital
Sykehusveien 25
1478 Lørenskog, Norway
Tel: +47 928 67 976
E-mail: torbjorn.wisloff@ahus.no

SPONSOR SIGNATURE PAGE

Title: Akershus Clinical Trial (ACT) 1: Retrospective study to assess whether automatic safety monitors that present real-time electronic health data can improve clinical and patient safety outcomes

Protocol ID no:

ClinicalTrials.gov Identifier:

Sponsor signatory approval

Øystein Mæland
CEO,
Akershus University Hospital

Signature *Date*

I hereby declare that this Protocol has been developed in compliance with ICH GCP and the applicable regulatory requirements:

PI signatory approval

I hereby declare that I will conduct the study in compliance with the Protocol, ICH GCP and the applicable regulatory requirements:

Name:

Title:

PI signature *Date*

PROTOCOL SYNOPSIS

Intervention	Use of patient safety monitor providing real-time electronic health data
Protocol no.	1
Study title	Akershus Clinical Trial (ACT) 1: Retrospective study to assess whether patient safety monitors that present real-time electronic health data can improve clinical and patient safety outcomes
Sponsor	Akershus University Hospital, Norway
Responsible contact person	**Inge Skråmm**<br>MD, PhD<br>Division of Orthopedics<br>Akershus University Hospital<br>Sykehusveien 25<br>1478 Lørenskog, Norway<br>Tel: +47 922 12 722<br>E-mail: inge.skramm@ahus.no
Funding source(s)	Akershus University Hospital
Study center	Akershus University Hospital
Planned number of patients	Not applicable: retrospective design
Timelines	Estimated study start (first control): January 1, 2019<br>Estimated recruitment end (last prospective control): December 31, 2020<br>Estimated study start (first patient): January 1, 2021<br>Estimated recruitment end (last prospective patient): December 31, 2022<br>Follow-up period end date (last patient [LP] off study): December 31, 2023<br>End of study: January 1, 2028
Background and rationale	Patient injuries and adverse events are recognized as main challenges in the Norwegian National Action Plan for Patient Safety and Quality Improvement. The Norwegian Health Directory´s patient safety program "In safe hands 24/7" recommends huddle board as a tool to monitor patient safety measures, as defined in the program. However, there is limited information in the literature whether huddle boards impact clinical and patient safety outcomes. In addition, electronic health records now provide opportunity to integrate data for individual patients and report these back in structured format to clinical personnel on large screens, so-called Patient Safety Monitors (PSMs). Hence, we hypothesized that the use of PSMs in hospital wards in a Norwegian Orthopedic Department will reduce hospital length of stay and improve clinical and patient safety outcomes.
Study objectives	<u>Primary</u><br>To determine whether the use of PSM will reduce hospital length of stay (hours) compared to historical control patients.<br><br><u>Secondary</u><br>• To determine whether the use of PSM will reduce 30-day re-admission rate for any cause compared to historical control patients<br>• To determine whether the use of PSM will reduce 30-day all-cause mortality compared to historical control patients

	• To determine whether the use of PSM will reduce 1-year re-admission rate for any cause compared to historical control patients<br>• To determine whether the use of PSM will reduce 1-year all-cause mortality compared to historical control patients<br>• To determine whether the use of PSM will reduce unplanned ICU/CCU admissions during the index hospitalization compared to historical control patients<br>• To determine whether the use of PSM will increase nutritional screening within 24 h and documented in the Electronic Health Record (EHR) after admission compared to historical control patients<br>• To determine whether the use of PSM will increase the occurrence of nutritional support measures in the treatment plan documented in the EHR compared to historical control patients<br>• To determine whether the use of PSM will increase fall screening documented in the EHR within 24 h after admission compared to historical control patients<br>• To determine whether the use of PSM increases the number of patients subjected to National Early Warning Score (NEWS) 2 assessment documented in the EHR compared to historical control patients<br>• To determine whether the use of PSM increases the total number of NEWS 2 assessments documented in the EHR during in patients the index hospitalization compared to historical control patients<br>• To determine whether the use of PSM increases the prevalence of in-hospital anti-coagulation therapy compared to historical control patients<br>• To determine whether the use of PSM increases the prevalence of patients receiving anti-coagulation therapy at discharge from the index hospitalization compared to historical control patients<br>• To estimate total resource use during hospitalization for intervention and control patients<br>• To estimate 30-day cost for intervention and control patients, including current admission and readmissions
Study design	Single-center, pragmatic, pseudo-randomized controlled trial with *in silico*-control group for a non-pharmacological intervention<br>Screening phase: Not applicable<br>Study phase: All patients hospitalized at the dedicated wards within the study period
Patient population	<u>Inclusion</u><br>• Patients ≥18 years old<br>• Control group: All patients hospitalized at the Orthopedic Department, area A and B, wards S105 and S205 from Jan 1, 2019, to Dec 31, 2020 (prior to implementation of PSM)<br>• Intervention group: All patients hospitalized at the Orthopedic Department, area A and B, wards S105 and S205 from Jan 1, 2021, to Dec 31, 2022 (after implementation of PSM)<br>• Parallel control arm: Same analysis for area C and D, wards S105 and S205 for period Jan 1, 2019, to Dec 31, 2020, compared to period Jan 1, 2021, to Dec 31, 2021 (no implementation of PSM in neither period)

	<u>Exclusion</u><br>Previously included into the study (in case of patients presenting with a second hospitalization during the study period)
Visit schedule and assessments	We will use the data warehouse at Akershus University Hospital (Ahus) to perform the study. Data will be obtained from the health record systems like DIPS and Metavision and merged, structured, and presented via Ahus data warehouse. After regulatory approvals, data will be transferred to a secure data solution, i.e. Services for sensitive data (TSD) at UiO, and we will perform statistical analysis according to the statistical analysis plan.<br><br>The Division of Orthopedic Surgery at Ahus consists of two 7-days posts, S105 and S205, with four areas each: area A, B, C and D. We implemented PSM at area A and B at ward S105 and S205 in 2021 and will include patients hospitalized until December 31, 2022 (intervention group). To obtain a control group with similar patient composition and number of patients, we will compare the results for the intervention group with an *in silico*-control group, which is patients admitted to area A and B at ward S105 and S205 in 2019 and 2020 (prior to implementation of PSM). There was not given instructions in changes or other focus on identification and follow-up on the defined patient safety measures, except for the implementation of PSM. We will calculate Charlton Comorbidity Index to explore any major differences in patient composition for area A and B in 2019-2020 compared to 2021-2022.<br><br>In addition, PSM was not implemented in area C and D at S105 and S205 before March 2022. Given significant differences between the two periods for areas A and B, we will perform the same analyses for patients hospitalized at area C and D comparing the period 2019-2020 with 2021. Any difference seen also in areas C and D will not be a result of PSM as these groups never have used PSM.<br><br>We will apply to the Regional Ethics Committee, the Norwegian Directorate of Health, and the local Data Protection Officer to void the requirement for individual patient consent.
Data management and statistical analysis	This is a single-center, retrospective, pseudo-randomized controlled trial with *in silico*-control group studying the effect of PSM in an Orthopedic Department.<br><br>Please see separate statistical analysis plan (SAP).<br><br>The data will be summarized with respect to demographic and baseline characteristics, efficacy observations and measurements, and safety observations and measurements. Categorical data will be presented as absolute frequencies and percentages. For continuous data, N, mean (+SD) or median (interquartile range), will be presented. The analyses will be conducted on all patient data at the time of inclusion of the last patient.<br><br>The primary end-point is hospital length of stay (hours). Secondary end-points relate to, but are not limited to, 30-day readmission or all-cause mortality rates, need for ward in ICU/CCU, and nutritional screening. Analyses will be performed with appropriate choices of regression controlling for potential confounders, as descriped in the SAP. Time-to-event outcomes will also be presented in Kaplan-Meier plots with separate lines for each treatment group.<br><br>Financial resource utilization will be estimated by collecting information on hospital length of stay and type of hospital ward (ICU/CCU vs. standard) and by collecting information on all supplementary tests performed during or in conjunction with the index hospitalization.

	Patient follow-up and adjudication of clinical events during follow-up will be performed by either linking our data to national registries or by reviewing patient medical records or direct contact to the patient, next-in kin, or other health care providers.<br><br>Sample size calculations are not applicable for this study as we use retrospective design. However, we have calculated power of the data to demonstrate a potential change on the primary endpoint. Data from the Norwegian Patient Registry indicates that the mean length of stay in orthopedic wards is 4 days (standard deviation 2.5 days). We define a clinically relevant reduction in hospital length of stay to be at least 1 day (24 hours). Based on these numbers and expecting 1:1 ratio between hospitalized patients during the control period (2019-2020) and implementation period (2021-2022), we will need a minimum of 100 patients in the intervention arm and 100 patients in the in silico-control arm to have >80% probability to detect a difference with significance level 0.05. We know that >500 unique patients were hospitalized in the designated areas related to the study population, hence, we should have sufficient statistical power to detect a possible difference on the primary endpoint after implementation of PSM.

TABLE OF CONTENTS

CONTACT DETAILS 2
SPONSOR SIGNATURE PAGE 4
PROTOCOL SYNOPSIS 5
TABLE OF CONTENTS 9
LIST OF ABBREVIATIONS AND DEFINITIONS OF TERMS 11
1 INTRODUCTION 12
1.1 Background 12
1.2 Summary of Previous Clinical Studies and Current Strategies 12
1.3 Rationale for the Study 12
2 STUDY OBJECTIVES AND RELATED ENDPOINTS 12
2.1 Primary Endpoint 13
2.2 Secondary Endpoints 13
3 STUDY POPULATION 13
3.1 Selection of Study Population 13
3.2 Inclusion Criteria 14
3.3 Exclusion Criteria 14
3.4 Patient registration 14
4 STUDY EXECUTION 14
4.1 Patient Screening, Inclusion, and Randomization 14
4.2 Intervention 15
4.3 Patient Follow-Up During the Index Hospitalization and Data Storage 15
4.4 Adjudication of Diagnosis and Clinical Events 15
4.5 Quality of Life and Resource Utilization 15
5 DATA MANAGEMENT AND STATISTICAL ANALYSIS 15
5.1 Case Report Forms (CRFs) 15
5.2 Source Data 15
5.3 Source Data Verification 15
5.4 Storage of Study Documentation 16
5.5 Study Design 16
5.6 Sample Size & Statistical Considerations 16
5.7 Safety Analysis 17
5.8 Interim Analysis 17
6 ETHICAL AND REGULATORY REQUIREMENTS 17

6.1 Ethical Considerations 17
6.1.2 Void of Informed Consent 17
6.2 Obligations of Investigators 18
6.3 Record Retention 19
6.4 Audits 19
6.5 Publication Policy 19
7 STUDY MANAGEMENT 19
7.1 Investigator Delegation Procedure 19
7.2 Study Amendments 19
7.3 Audit and Inspections 19
8 TRIAL SPONSORSHIP AND FINANCING 20
9 TRIAL INSURANCE 20
10 ACKNOWLEDGEMENTS 20
APPENDIX ..... ERROR! BOOKMARK NOT DEFINED.
REFERENCES 21

LIST OF ABBREVIATIONS AND DEFINITIONS OF TERMS

Abbreviation or special term	Explanation
PSM	Patient Safety Monitor
ICC/CCU	Intensive Care Unit/Critical Care Unit
EHR	Electronic Health Record
NEWS 2	National Early Warning Score 2
Ahus	Akershus University Hospital
DIPS	Distributed Information and Patient Data System in Hospitals (EHR system)
UiO	University of Oslo
TSD	Services for Sensitive Data

1 INTRODUCTION

1.1 Background

Patient safety and prevention of adverse events through quality improvements have been focus areas for most Western health care systems the last decade. Higher expectations, an aging population with increasing co-morbidities, and high workload for hospital staff can contribute to unsafe work environments and make safety initiatives imperative. In Norway, the National Action Plan for Patient Safety and Quality Improvement for 2019-2023 states that patient injuries and adverse events are main challenges for the health system [1]. Accordingly, the Action Plan for Patient Safety and Quality Improvement promotes the safety program “In safe hands 24/7”. One of the recommendations of the Action Plan is to implement brief multidisciplinary meetings around huddle boards. During these meetings, there is focus on routines to prevent pressure ulcers and falls and to identify patients at high risk of adverse events. Limited information is available on the clinical value from huddle boards. In addition, electronic health records now provide opportunity to integrate data for individual patients and report these back in structured format to clinical personnel on large screens, so-called Patient Safety Monitors (PSMs). Analogous to the situation for huddle boards, no information is available on whether PSM may improve clinical and patient safety outcomes. Accordingly, in this study we hypothesized that use of PSMs in hospital wards in a Norwegian Orthopedic Department will reduce hospital length of stay (primary outcome) and improve clinical and patient safety outcomes (secondary outcomes).

1.2 Summary of Previous Clinical Studies and Current Strategies

Literature regarding the implementation of PSM is limited and most prior work has been limited to analog huddle boards. In the UK, a report of the Huddle Up for Safer Healthcare (HUSH) project [2] demonstrated that implementation of patient safety huddles (PSH) in 64 hospital wards improved teamwork, provided a feeling of safer work environment for nurses and improved overall safety grade of the wards. Similarly, experience from the implementation of the Situation Awareness For Everyone (SAFE) safety improvement programme in pediatric wards in UK, which also included daily huddle board meetings, was enhanced awareness of important factors related to patient safety indices. Moreover, huddle board meetings were reported to improve teamwork and efficiency in the hospital wards [3]. To the best of our knowledge, no information is currently available on the effect of PSM on clinically relevant endpoints like hospital length of stay or adverse events.

1.3 Rationale for the Study

PSMs are currently being implemented in a variety of forms across most Western health care systems and it is important to document possible effects on clinical and patient safety endpoints.

2 STUDY OBJECTIVES AND RELATED ENDPOINTS

Patient injuries and adverse events are recognized as main challenges in the Norwegian National Action Plan for Patient Safety and Quality Improvement. The Norwegian Health Directory´s patient safety program "In safe hands 24/7" recommends huddle board as a tool to monitor patient safety measures, as defined in the program. However, currently limited information is available in the literature on the clinical value from huddle boards. In addition, electronic health records now provide opportunity to integrate data for individual patients and report these back in structured format to clinical personnel on large screens, so-called Patient Safety Monitors (PSMs). The aim of this retrospective, pseudo-randomized controlled single center non-pharmacological trial with *in silico*-control group is to assess whether implementation of PSM in a Norwegian Orthopedic Department can reduce hospital length of stay and improve clinical and patient safety outcomes.

2.1 Primary Endpoint

- To determine whether the use of PSM will reduce hospital length of stay (hours) compared to historical control patients

2.2 Secondary Endpoints

- To determine whether the use of PSM will reduce 30-day re-admission rate for any cause compared to historical control patients
- To determine whether the use of PSM will reduce 30-day all-cause mortality compared to historical control patients
- To determine whether the use of PSM will reduce 1-year re-admission rate for any cause compared to historical control patients
- To determine whether the use of PSM will reduce 1-year all-cause mortality compared to historical control patients
- To determine whether the use of PSM will reduce the number of ICU/CCU admissions during the index hospitalization compared to historical control patients
- To determine whether the use of PSM will increase nutritional screening within 24 h and documented in the Electronic Health Record (EHR) after admission compared to historical control patients
- To determine whether the use of PSM will increase the occurrence of nutritional support measures in the treatment plan documented in the EHR compared to historical control patients
- To determine whether the use of PSM will increase fall screening documented in the EHR within 24 h after admission compared to historical control patients
- To determine whether the use of PSM increases the number of patients subjected to National Early Warning Score (NEWS) 2 assessment documented in the EHR compared to historical control patients
- To determine whether the use of PSM increases the total number of NEWS 2 assessments documented in the EHR during in patients the index hospitalization compared to historical control patients
- To determine whether the use of PSM increases the prevalence of anti-coagulation therapy compared to historical control patients
- To determine whether the use of PSM increases the prevalence of patients receiving anti-coagulation therapy at discharge from the index hospitalization compared to historical control
- To estimate total resource use during hospitalization for intervention and control patients
- To estimate 30-day cost for intervention and control patients, including current admission and readmissions
- To determine whether the use of PSM increases the prevalence of medication reconciliation compared to historical control patients.

3 STUDY POPULATION

3.1 Selection of Study Population

- Control group: All patients hospitalized at the Orthopedic Department, area A and B, wards S105 and S205 from January 1, 2019, to Dec 31, 2020 (prior to implementation of PSM)
- Intervention group: All patients hospitalized at the Orthopedic Department, area A and B, wards S105 and S205 from Jan 1, 2021, to Dec 31, 2022 (after implementation of PSM)
- Parallel control arm: Same analysis for area C and D, wards S105 and S205 for period Jan 1, 2019, to Dec 31, 2020, compared to period Jan 1, 2021, to Dec 31, 2021 (no implementation of PSM in neither period)

3.2 Inclusion Criteria

All the following conditions must apply to the prospective patient at screening prior to inclusion in the study:

- Age ≥18 y
- Admitted to the designated areas during the study period

3.3 Exclusion Criteria

Patients will be excluded from the study if they meet any of the following criteria:

- Previously included into the study (in case of patients presenting with a second hospitalization during the study period)

3.4 Patient registration

Each patient in the study will be uniquely identified by a code starting with "PSM", followed in numerical order from 1 up to the last patient that is included in the study. Once assigned to a patient, a patient number will not be re-used.

4 STUDY EXECUTION

4.1 Patient Screening, Inclusion, and Randomization

We will only include patients hospitalized at the designated areas at Ahus, location Lørenskog, during the study period. To extract data, we will use the data warehouse at Ahus. Data will be obtained from EHR systems like DIPS and Metavision and the data will be merged, structured, and presented via Ahus data warehouse. After regulatory approvals, data will be transferred to a secure data solution, i.e. TSD at UiO, and we will perform statistical analysis according to the statistical analysis plan.

The Division of Orthopedic Surgery at Ahus consists of two 7-days wards, S105 and S205, with four areas each: area A, B, C and D. We implemented PSM at area A and B at ward S105 and S205 in January 2021. The interventions group will contain patients hospitalized at wards with PSM implemented at the Division of Orthopedic Surgery from January 2021 to December 2022. To obtain a control group with similar patient composition, we will compare the results for the intervention group with an *in silico*-control group, which is patients admitted to area A and B at ward S105 and S205 in 2019-2020 (prior to implementation of PSM). There was not given instructions in changes or other focus on identification and follow-up on the defined patient safety measures, except for the implementation of PSM. We will calculate Charlton Comorbidity Index to explore any major differences in patient composition for area A and B in the periods 2019-2020 versus 2021-2022.

In addition, PSM was not implemented in area C and D at S105 and S205 before March 2022. Given significant differences between the two periods for areas A and B, we will perform the same analyses for patients hospitalized at area C and D comparing the period 2019-2020 with 2021. Any difference seen also in areas C and D will not be a result of PSM as these groups never have used PSM.

We will apply to the Regional Ethics Committee and the local Data Protection Officer to void requirement for individual patient consent.

4.2 Intervention

Intervention: PSM implementation at two orthopedic wards.

Control: Standard of care in orthopedic wards without PSM.

4.3 Patient Follow-Up During the Index Hospitalization and Data Storage

Data extraction will be performed by the Department of Analysis, Akershus University Hospital according to their regulatory approvals and by standard operating procedures for data extraction in the Ahus data warehouse. The data will be stored on safe serves, first at Akershus University Hospital and later transferred to TSD, University of Oslo. All statistical analysis will be performed at TSD.

4.4 Adjudication of Diagnosis and Clinical Events

We will not perform adjudication for this study.

4.5 Costs and Resource Utilization

We will combine data on in-hospital and 30-day resource use to estimate total 30-day cost for intervention and control patients. Data will be based on estimated costs per patient as calculated in the hospital system. Resource consumption will be reported both for the index stay, and for additional contacts with the hospital during the first 30 days after discharge. In addition to reporting of total costs, we will perform regression analysis to estimate difference in costs between intervention and control using a generalized linear model, controlling for potential counfounders.

5 DATA MANAGEMENT AND STATISTICAL ANALYSIS

5.1 Case Report Forms (CRFs)

Not applicable to this study as we will use a retrospective design with all data recorded in the Electronic Health Records of the individual patients. We will use the data warehouse at Ahus to extract from the systems DIPS and Metavision and we will present merged and structured data via Ahus data warehouse. After regulatory approvals, data will be transferred to a secure data solution, i.e. TSD at UiO, and we will perform statistical analysis according to the statistical analysis plan. Patient Id will be anonymized prior to transfer of data to TSD, and all statistical analysis will be performed on aggregated, anonymous data.

5.2 Source Data

The Electronic Health Records of individual patients.

5.3 Source Data Verification

Not applicable.

5.4 Storage of Study Documentation

The investigator shall arrange for the retention of the patient identification and the code list. Patient files shall be kept for the maximum period permitted by each hospital. The study documentation shall be retained and stored during the study and for 5 years after study closure. All information concerning the study will be stored in a safe place inaccessible to unauthorized personnel.

5.5 Study Design

This is a single center, randomized controlled trial with *in silico*-control group of a non-pharmacological intervention.

The data will be summarized with respect to demographic and baseline characteristics, efficacy observations and measurements, and safety observations and measurements. Categorical data will be presented as absolute frequencies and percentages. For continuous data, N, mean±SEM (parametric data) or median (quartile 1-3) (non-parametric data), and range may be presented. Time to event variables and Kaplan-Meier product-limit estimates will be presented stratified by intervention group. Risk of events in time-to-event models will be assessed by unadjusted and adjusted Cox regression. The analyses will be conducted on all patient data at the time of inclusion of the last patient. Financial resource utilization will be performed by collecting information on hospital length of stay and type of hospital ward (ICU/CCU vs. standard) and by collecting information on all supplementary tests performed during or in conjunction with the index hospitalization.

The primary end-point is hospital length of stay (hours). Secondary end-points relate to, but are not limited to, 30-day readmission or all-cause mortality rates, need for ward in ICU/CCU, and nutritional screening.

Patient follow-up and adjudication of clinical events during follow-up will be performed by either linking our data to national registries or by reviewing patient medical records or direct contact to the patient, next-in kin, or other health care providers.

5.6 Sample Size & Statistical Considerations

Sample size calculations are not applicable for this study as we use retrospective design. However, we have calculated power of the data to demonstrate a potential change on the primary endpoint. Data from the Norwegian Patient Registry indicates that the mean length of stay in orthopedic wards is 4 days (standard deviation 2.5 days). We define a clinically relevant reduction in hospital length of stay to be at least 1 day (24 hours). Based on these numbers and expecting 1:1 ratio between hospitalized patients during the control period (2019-2020) and implementation period (2021-2022), we will need a minimum of 100 patients in the intervention arm and 100 patients in the in silico-control arm to have >80% probability to detect a difference with significance level 0.05. We know that >500 unique patients were hospitalized in the designated areas related to the study population, hence, we should have sufficient statistical power to detect a possible difference on the primary endpoint after implementation of PSM.

For details concerning statistical analyses, please see separate statistical analysis plan (SAP).

The data will be summarized with respect to demographic and baseline characteristics, efficacy observations and measurements, and safety observations and measurements. Categorical data will be presented as absolute frequencies and percentages. For continuous data, N, mean (+SD) or median (interquartile range), will be presented. The analyses will be conducted on all patient data at the time of inclusion of the last patient.

The primary end-point is hospital length of stay (hours). Secondary end-points relate to, but are not limited to, 30-day readmission or all-cause mortality rates, need for ward in ICU/CCU, and nutritional screening. Analyses will be performed with appropriate choices of regression controlling for potential confounders, as descriped in the SAP. Time-to-event outcomes will also be presented in Kaplan-Meier plots with separate lines for each treatment group.

5.7 Safety Analysis

No safety analysis will be performed.

5.8 Interim Analysis

We will not perform interim analysis.

6 ETHICAL AND REGULATORY REQUIREMENTS

6.1 Ethical Considerations

6.1.1 General Considerations

The responsible investigator will ensure that this study is conducted in agreement with the Declaration of Helsinki (Tokyo, Venice, Hong Kong, Somerset West and Edinburgh amendments) and the laws and regulations of the country where the trial is performed. The protocol has been written, and the study will be conducted according to the ICH Harmonized Tripartite Guideline for Good Clinical Practice (ref: http://www.ifpma.org/ethics/ifpma-code-of-practice/about-ifpma-code-of-practice.html). The study will be evaluated by the Regional Ethics Committee and other government agencies before initiation. The protocol will be registered in www.clinicaltrials.gov before inclusion of the first patient.

6.1.2 Void of Informed Consent

The proposed study will be conducted using data already collected for clinical purposes. The project will be considered and evaluated by the Regional Ethics Committee and the Data Protection Officer at Ahus before study initiation. Ahus is the project owner and responsible for all research data.

We will request a waiver for patient consent in this study. We are aware of the strict rules and that all requirements in Helsepersonelloven §29 must be met before approval of waiver for patient consent.

Quality improvement

In the current project, we aim to compare outcomes before and after the implementation of PSM at the Division of Orthopedic Surgery at Ahus. The results from the project will evaluate current practices at Ahus and is *quality improvement.*

Difficulty in acquiring informed consent

The project group has discussed potential possibilities, challenges, and barriers of collecting informed consent from the patients. We know that a large proportion of eligible patients admitted to the Division of Orthopedic Surgery are elderly and will have passed away since hospital admission. Further, many of the surviving elderly patients have various degrees of cognitive impairment, and exclusion due to lack of informed consent would preclude generalization of the results to this large and vulnerable patient group. Both these factors will complicate the matters of retrospective consent even more. Selection bias in retrospective studies strongly undermines the external validity of the results, and it is crucial to have a complete patient population to avoid such confounding. Previous reports have documented significant differences between participants and non-participants in observational studies that require consent for use of data from medical records, largely threatening the validity study results. With the requirement of informed consent, we worry deeply that our results will not answer the study hypotheses correctly, and wrongfully inform us about the effects of PSM at Ahus. Regarding dissemination of the study results and information to be provided where personal data have not been obtained from the data subject, we will thoroughly inform the public through lectures for patient organizations and at popular science meetings at Ahus (annually conducted "Åpen dag - fokus på fag og forskning"). We will publish our results on the web pages of Ahus, forskning.no, and the University of Oslo, fulfilling possible information requirements.

The research in question is of significant interest to society

The present project is important as a quality improvement project at Ahus and will significantly increase our knowledge on the effects of PSM in one of the largest hospitals in Norway. There is currently lack of evidence on the effects of such interventions, and this knowledge is crucial for the assessment of current national patient safety programs. Inclusion of patients without a retrospectively informed consent will make it possible to use this set of data in a very short time, and make the findings immediately available to decision makers, the academic community, and the general population instead of waiting for many years to prospectively collect a sample of similar size.

The participants' welfare and integrity are ensured

The study will solely use data already collected for clinical purposes. No additional or especially sensitive data except for routine data from electronic hospital records and compulsory national health registries will be collected. No new interventions are introduced. All data will be handled according to the current data security guidelines from Ahus and the General Data Protection Regulation and will be stored on encrypted and restricted research servers at Akershus University Hospital and the University of Oslo. Ahus has a local data warehouse that enables automatic extraction of clinical data to a study database, largely eliminating the need to access individual patient electronic hospital records. After the automatized data extraction by the data warehouse, acquisition of remaining necessary study data from electronic hospital records will be performed by a restricted group of study collaborators, maximizing privacy and data protection. The study database will be anonymized before analyses and the scrambling key stored in a separate encrypted and secure server location.

All the analyses in the current project will be performed on previously collected data, and none of the results will have any consequence for treatment or other medical follow-up for the individual patient. All results will be presented on a group level without risk of backtracking the identity of study participants. Altogether, the proposal does not raise any serious ethical issues limiting its feasibility.

6.2 Obligations of Investigators

The Principal Investigator is responsible for the different parts of the study, including obtaining approvals, extracting data from patient system via Ahus data warehouse, and performing statistical analysis.

6.3 Record Retention

Study documentation includes data entered into the trial database prior to statistical analysis. All other data will be stored as part of the patient's normal electronic health records.

Government agency regulations and directives require that the study investigator must retain all study documentation pertaining to the conduct of a clinical trial. Study documents should be kept on file for 5 years after the completion and final study report.

6.4 Audits

Not applicable as this is a retrospective study.

6.5 Publication Policy

The findings of this study will be published independent of its outcome. All personnel who have contributed significantly with the planning or to perform the study (Vancouver convention 1988) may be included in the list of authors.

7 STUDY MANAGEMENT

7.1 Investigator Delegation Procedure

The Principal Investigator is responsible for making and updating a "delegation of tasks" listing all the involved co-workers and their role in the project. He will ensure that appropriate training relevant to the study is given to all of these staff, and that any new information of relevance to the performance of this study is forwarded to the staff involved.

7.2 Study Amendments

If it is necessary for the study protocol to be amended, the amendment and/or a new version of the study protocol (Amended Protocol) must be notified to and approved by the Competent Authority and the Ethics Committee according to EU and national regulations.

7.3 Audit and Inspections

Authorized representatives of a regulatory authority and Ethics Committee may visit the center to perform inspections, including source data verification. Likewise the representatives from the sponsor may visit the center to perform an audit. The purpose of an audit or inspection is to systematically and independently examine all study-related activities and documents to determine whether these activities were conducted, and data were recorded, analyzed, and accurately reported according to the protocol, Good Clinical Practice (ICH GCP), and any applicable regulatory requirements. The Principal Investigator will ensure that the inspectors and auditors will be provided with access to source data/documents.

8 TRIAL SPONSORSHIP AND FINANCING

The study is sponsored by Ahus.

9 TRIAL INSURANCE

This study is covered by the general insurance of Ahus.

10 ACKNOWLEDGEMENTS

HR and KB have drafted most of the study protocol. JAG, OL, LÅM, and IS were responsible for development and clinical implementation of PSM and participated in drafting and revising the protocol. The other co-authors have also contributed to the development of the final study protocol.

REFERENCES

1. Helsedirektoratet. *Nasjonal handlingsplan for pasientsikkerhet og kvalitetsforbedring*. 2019; Available from: https://www.helsedirektoratet.no/veiledere/ledelse-og-kvalitetsforbedring-i-helse-og-omsorgstjenesten/Nasjonal%20handlingsplan%20for%20pasientsikkerhet%20og%20kvalitetsforbedring%202019-2023.pdf.
2. Lamming, L., et al., *Fidelity and the impact of patient safety huddles on teamwork and safety culture: an evaluation of the Huddle Up for Safer Healthcare (HUSH) project.* BMC Health Serv Res, 2021. **21**(1): p. 1038.
3. Stapley, E., et al., *Factors to consider in the introduction of huddles on clinical wards: perceptions of staff on the SAFE programme.* Int J Qual Health Care, 2018. **30**(1): p. 44-49.
