# Supplementary material for: Pragmatic, quasi-experimental, pseudo-randomized clinical trial to assess the impact of patient safety monitors on clinical and patient safety outcomes: The Akershus Clinical Trial (ACT) 1
Source: PLoS One. 2025 Oct 22;20(10):e0335052. doi: 10.1371/journal.pone.0335052 (PMC12543108; doi:10.1371/journal.pone.0335052)
Supplement: Attachment — Submitted filename: ACT1 PatientSafety Response to Reviewers.docx [file pone.0335052.s008.docx]

**Academic Editor (Comments for the Author):**

Journal Requirements:

When submitting your revision, we need you to address these additional requirements.

1. Please ensure that your manuscript meets PLOS ONE's style requirements, including those for file naming. The PLOS ONE style templates can be found at

<https://journals.plos.org/plosone/s/file?id=wjVg/PLOSOne_formatting_sample_main_body.pdf> and

[https://journals.plos.org/plosone/s/file?id=ba62/PLOSOne_formatting_sample_title_authors_affiliations.pdf](https://track.editorialmanager.com/CL0/https:%2F%2Fjournals.plos.org%2Fplosone%2Fs%2Ffile%3Fid=ba62%2FPLOSOne_formatting_sample_title_authors_affiliations.pdf/1/010f01982c534322-c1165aa2-4b1f-45af-b866-a28d73621516-000000/RjJRZHvdgzOOc9wkwrxl4pTEVAL1reV3K16b2_n3O5I=220).

**Our response:** We appreciate the clear guidance on formatting. The manuscript has now been revised to fully comply with PLOS ONE’s style requirements, as shown in the attached track changes version.

2. In the online submission form, you indicated that [Data will be make available upon reasonable request.].

All PLOS journals now require all data underlying the findings described in their manuscript to be freely available to other researchers, either 1. In a public repository, 2. Within the manuscript itself, or 3. Uploaded as supplementary information.

This policy applies to all data except where public deposition would breach compliance with the protocol approved by your research ethics board. If your data cannot be made publicly available for ethical or legal reasons (e.g., public availability would compromise patient privacy), please explain your reasons on resubmission and your exemption request will be escalated for approval.

**Our response:** Unfortunately, the dataset used in this study is not publicly available, as the Data Protection Authority approval and patient consent do not allow for such publication. However, the study group welcomes initiatives for cooperation, and data access may be granted upon application. The updated data availability statement in the manuscript now reads:

*“The dataset underlying this study contains sensitive patient information and cannot be made publicly available, as the approval from the Norwegian Data Protection Authority and patient consent do not permit open sharing of individual-level data. De-identified data may be made available upon reasonable request for researchers who meet the criteria for access to confidential data. Data requests can be directed to the Akershus University Hospital Clinical Research Unit (ACR), which serves as the long-term institutional data custodian (contact: acr@ahus.no).”*

3.  Please include captions for your Supporting Information files at the end of your manuscript, and update any in-text citations to match accordingly. Please see our Supporting Information guidelines for more information: <http://journals.plos.org/plosone/s/supporting-information>.

**Our response:** This has been corrected. Supporting Information files now have proper captions, and all in-text citations have been updated to match PLOS ONE style, which now reads as follows:

***“Supporting information***

***S1 Table. Baseline characteristics for bays where patient safety monitors were not implemented****. *p-value for difference between intervention and control group, t-test for age, Chi-square test otherwise.*

***S2 Table. Odds ratio (OR) for safety endpoint in the period after implementation of patient safety monitors (PSM) compared to before for intervention wards and control wards (where PSM was not implemented).*** *All analyses adjusted for age, sex, Charlson comorbidity score, cause of index admission, and admission period (2019–2020 vs. 2021–2022).*

***S3 Table. Number of NEWS scores taken during the index hospital stay for the intervention wards and control wards (where PSM was not implemented).*** *All analyses adjusted for age, sex, Charlson comorbidity score, cause of index admission, and admission period (2019–2020 vs. 2021–2022).*

***S4 Table. Time-to-event analyses.*** *All analyses adjusted for age, sex, Charlson comorbidity score, cause of index admission, and admission period (2019–2020 vs. 2021–2022).*

***S1 Appendix. Predefined study protocol.***

***S2 Appendix. Predefined statistical analysis plan.***

***S3 Appendix. Signed protocol for internal monitoring of data extraction in the study****.”*

4.  Thank you for stating the following in the Competing Interests section:

[KB has received speaker honoraria from Boehringer Ingelheim, Novartis, Amgen, and AstraZeneca.].

We note that one or more of the authors are employed by a commercial company: name of commercial company.

1. Please provide an amended Funding Statement declaring this commercial affiliation, as well as a statement regarding the Role of Funders in your study. If the funding organization did not play a role in the study design, data collection and analysis, decision to publish, or preparation of the manuscript and only provided financial support in the form of authors' salaries and/or research materials, please review your statements relating to the author contributions, and ensure you have specifically and accurately indicated the role(s) that these authors had in your study. You can update author roles in the Author Contributions section of the online submission form.

Please also include the following statement within your amended Funding Statement.

“The funder provided support in the form of salaries for authors [insert relevant initials], but did not have any additional role in the study design, data collection and analysis, decision to publish, or preparation of the manuscript. The specific roles of these authors are articulated in the ‘author contributions’ section.”

If your commercial affiliation did play a role in your study, please state and explain this role within your updated Funding Statement.

2. Please also provide an updated Competing Interests Statement declaring this commercial affiliation along with any other relevant declarations relating to employment, consultancy, patents, products in development, or marketed products, etc.

Within your Competing Interests Statement, please confirm that this commercial affiliation does not alter your adherence to all PLOS ONE policies on sharing data and materials by including the following statement: ""This does not alter our adherence to  PLOS ONE policies on sharing data and materials.” (as detailed online in our guide for authors <http://journals.plos.org/plosone/s/competing-interests>) . If this adherence statement is not accurate and  there are restrictions on sharing of data and/or materials, please state these. Please note that we cannot proceed with consideration of your article until this information has been declared.

Please include both an updated Funding Statement and Competing Interests Statement in your cover letter. We will change the online submission form on your behalf.

**Our response:** We appreciate this clarification. As requested, we have provided updated Funding and Competing Interests Statements in the cover letter. In brief, KB has received speaker honoraria from Boehringer Ingelheim, Novartis, Amgen, and AstraZeneca. None of these companies provided funding, salaries, or had any role in the study design, data collection and analysis, decision to publish, or preparation of the manuscript. This does not alter our adherence to PLOS ONE policies on sharing data and materials.

5. If the reviewer comments include a recommendation to cite specific previously published works, please review and evaluate these publications to determine whether they are relevant and should be cited. There is no requirement to cite these works unless the editor has indicated otherwise. 

**Our response:** We note the editor’s comment. Neither of the reviewers suggested specific publications to be cited, and no additional references were therefore added.

6. Please review your reference list to ensure that it is complete and correct. If you have cited papers that have been retracted, please include the rationale for doing so in the manuscript text, or remove these references and replace them with relevant current references. Any changes to the reference list should be mentioned in the rebuttal letter that accompanies your revised manuscript. If you need to cite a retracted article, indicate the article’s retracted status in the References list and also include a citation and full reference for the retraction notice.

**Our response:** The reference list has been carefully reviewed, and no retracted papers were identified. Reference 5 has been updated with an English translation of the title and corrected URL. The updated reference now reads:

*“5. Helsedirektoratet [Norwegian Directorate of Health]. Nasjonal handlingsplan for pasientsikkerhet og kvalitetsforbedring 2019–2023 [National action plan for patient safety and quality improvement 2019–2023]. Oslo; 2023. Available from: https://www.helsedirektoratet.no/veiledere/ledelse-og-kvalitetsforbedring-i-helse-og-omsorgstjenesten/Nasjonal%20handlingsplan%20for%20pasientsikkerhet%20og%20kvalitetsforbedring%202019-2023.pdf”*

Additional Editor Comments:

Thank you for submitting this manuscript evaluating the impact of PSM on patient outcomes. The topic is important and relevant to efforts aimed at improving hospital safety. After reviewing the manuscript, I would like to raise the following points:

**Our response:** We are grateful for the editor’s careful reading of our work and the constructive feedback. Responses to each specific point are provided below.

#1 Study Design and Protocol Registration: The study is described as a “pragmatic, pseudo-randomized trial,” but the allocation method suggests that the design is more accurately classified as a quasi-experimental study. Please consider revising this terminology to reflect the actual methodological approach. Additionally, please clarify whether the study protocol was prospectively registered. If not, this should be acknowledged as a limitation due to potential reporting bias.

**Our response:** We thank the editor for this important clarification. We agree that our study is best described as a quasi-experimental, pragmatic study, since allocation was not based on true randomization. Still, after careful consideration, we have retained the term “pseudo-randomized” in combination with “quasi-experimental” to reflect the allocation mechanism: patients were assigned to intervention or control depending on calendar year of admission (2019–2020 for control; 2021–2022 for intervention, after electronic whiteboards were implemented). This approach, although not truly random, mimics randomization and is supported by the highly balanced distribution of baseline characteristics between groups (S1 Table). If the editor considers the use of “pseudo-randomized” to be misleading, we are of happy to limit the terminology to “quasi-experimental” only. We have added a sentence in the Methods section (Study design) to further elaborate this:

*“Although allocation was calendar-based rather than truly random, this approach functions as a form of pseudo-randomization, since patient assignment was independent of individual characteristics and was therefore expected to yield balanced baseline characteristics between groups.”*

Regarding protocol registration, we confirm that the study was not prospectively registered in a public trial registry. However, both a study protocol and a statistical analysis plan were predefined, formally approved by the study group, and electronically time-stamped before study initiation. We have now clarified this in the Methods section, and the lack of prospective registration has been explicitly acknowledged as a limitation in the Discussion.

In the revised manuscript we have clarified the study design by consistently referring to it as a quasi-experimental, pragmatic, pseudo-randomized study. In addition, we have updated the Methods section to include the following sentence:

*“A predefined study protocol and statistical analysis plan were approved by the study group and electronically time-stamped prior to study initiation (S1 and S2 Appendices).”*

#2 Clinical Implications and Subgroup Effects: Although the overall findings did not demonstrate statistically significant benefits from the PSM intervention, it remains possible that specific patient subgroups, such as those at higher risk for delirium or falls, might benefit more clearly. I encourage the authors to consider performing subgroup analyses to explore PSM potential effects of certain groups.

**Our response:** We thank the editor for this valuable suggestion. We have carefully considered the possibility of conducting subgroup analyses. However, as outlined in our predefined statistical analysis plan, no such subgroup analyses were specified a priori. We believe that adding further post hoc analyses at this stage would not be consistent with the protocol-driven nature of the study and may risk overinterpretation.

Moreover, since the intervention (implementation of electronic safety boards) was delivered at the ward level, we consider the primary focus to be at the unit rather than the individual patient level, which limits the interpretability of subgroup analyses of specific patient populations.

That said, in response to the editor’s request, we have performed exploratory subgroup analyses stratified by age (<80 vs. ≥80 years) and, separately, by comorbidity burden (Charlson Comorbidity Index). These analyses did not reveal any significant interactions (see table below/attached). We are happy to provide these results as supplementary material if the editor considers this necessary for readers.

#3 Interpretation of Findings and Cost-Effectiveness Recommendation: The manuscript suggests that cost-effectiveness analysis (CEA) of the PSM intervention may be warranted. Given that the intervention did not show meaningful clinical benefit in the overall analysis, this recommendation appears premature. Please reconsider this suggestion and revise it to align more appropriately with the findings.

**Our response:** We agree that our original statement regarding cost-effectiveness may be premature given the neutral findings. Since PLOS ONE does not require a “Key Messages” section, we propose to remove this section altogether to avoid overstating implications and to align more closely with the journal’s format. If the editor prefers, we are happy to retain a shortened version with more cautious wording.

Reviewers' comments:

Reviewer's Responses to Questions

**Comments to the Author**

1. Is the manuscript technically sound, and do the data support the conclusions?

The manuscript must describe a technically sound piece of scientific research with data that supports the conclusions. Experiments must have been conducted rigorously, with appropriate controls, replication, and sample sizes. The conclusions must be drawn appropriately based on the data presented.

Reviewer #1: Yes

Reviewer #2: Yes

2. Has the statistical analysis been performed appropriately and rigorously?

Reviewer #1: Yes

Reviewer #2: Yes

3. Have the authors made all data underlying the findings in their manuscript fully available?

The [PLOS Data policy](http://track.editorialmanager.com/CL0/http:%2F%2Fwww.plosone.org%2Fstatic%2Fpolicies.action%23sharing/1/010f01982c534322-c1165aa2-4b1f-45af-b866-a28d73621516-000000/lHQn5V-mfys8qM-Xkx3b4GBWU9rlwG3aY2kBoIcayDo=220) requires authors to make all data underlying the findings described in their manuscript fully available without restriction, with rare exception (please refer to the Data Availability Statement in the manuscript PDF file). The data should be provided as part of the manuscript or its supporting information, or deposited to a public repository. For example, in addition to summary statistics, the data points behind means, medians and variance measures should be available. If there are restrictions on publicly sharing data—e.g. participant privacy or use of data from a third party—those must be specified.

Reviewer #1: Yes

Reviewer #2: Yes

4. Is the manuscript presented in an intelligible fashion and written in standard English?

PLOS ONE does not copyedit accepted manuscripts, so the language in submitted articles must be clear, correct, and unambiguous. Any typographical or grammatical errors should be corrected at revision, so please note any specific errors here.

Reviewer #1: Yes

Reviewer #2: Yes

**Our response:** We thank both reviewers for their thorough evaluation of the study.

5. Review Comments to the Author

Please use the space provided to explain your answers to the questions above. You may also include additional comments for the author, including concerns about dual publication, research ethics, or publication ethics. (Please upload your review as an attachment if it exceeds 20,000 characters)

Reviewer #1: A well written paper that evaluates the implementation of a Patient Safety Monitor on clinical outcomes in a pre-/post-study. Most outcomes do not change, but some do actually get worse. Impressively the study is conducted through COVID with few changes in baseline characteristics.

There are only a few minor revisions that I would suggest for the benefit of the understanding of international readers.

1. Could you kindly add a picture of the monitor: the design of the interface might have significant impact on usability and clinical translation of warning signals.

**Our response:** We appreciate this helpful suggestion. We agree that illustrating the user interface improves interpretability and have added an example illustration of the PSM (S1 Fig) to our manuscript.

*“****S1 Fig. Example screenshot of the Patient Safety Monitor (PSM) interface.*** *The electronic board displays patient-level information relevant to safety processes in real time. Columns include: patient ID (anonymized here), age, admission date, NEWS2 early warning score, peripheral venous catheter (PVC) status, medication reconciliation, physician contact time, prophylaxis (e.g. anticoagulation, antibiotics, osteoporosis therapy), allied health services (physiotherapy, social/municipal health services), discharge planning, nutritional screening, pressure ulcer screening, fall risk screening, and delirium screening (4AT). Color coding highlights overdue tasks (red), completed items (green), and intermediate status (orange). This screenshot illustrates the system in routine use on an orthopaedic ward.”*

2. I am not sure whether you mean 'bay' - a bay has usually got 4-6 beds, a ward has 20-30 beds. I would struggle to understand why units of 6 bays would have autonomy to implement or not implement? Why would this be?

**Our response:** Thank you for raising this point. In our hospital, the organisational unit is indeed the ward, which is further subdivided into several multi-bed bays (tuns), each typically hosting 6–10 patients. The PSMs were implemented gradually: in 2021 it was installed in two orthopaedic bays, while the remaining bays continued usual care. Two neighboring non-PSM bays served as a natural sensitivity analysis group, helping us to identify temporal trends not attributable to the intervention. We have clarified this in the Methods section (Patient population and PSM implementation in the orthopedic ward):

*“The study population consists of all patients admitted to two orthopedic bays (multi-bed subunits of the ward, typically hosting 6–10 patients each) at Akershus University Hospital between 01/01/2019 and 31/12/2022 who implemented PSM (DNV Imatris, Porsgrunn, Norway) starting 01/01/2021.”*

3. I don't think that this is a pseudo-randomised trial - it is a pre-post study with a parallel group?

**Our response:** We acknowledge this observation. As also noted by the editor, we agree that the study is best classified as a quasi-experimental, pragmatic trial. We have nevertheless retained the qualifier “pseudo-randomized” to reflect the calendar-based allocation mechanism, as explained in our response to the editor and clarified in the Methods section. If the editor ultimately finds this terminology misleading, we are of course willing to restrict it to “quasi-experimental” only.

4. I would value the view of the authors why some of the outcomes might have worsened. What other things happened at the time? Did teams get complacent given an over-reliance on new technology? Can you comment on bed occupancy and number of operations per year that might have been affected by COVID?

**Our response:** This is an important consideration. To address it, we have retrieved additional data on contextual factors that may have influenced the results, including patient throughput, nursing staffing, and surgical activity for the study periods 2019–2020 (control) and 2021–2022 (intervention). The number of elective operations decreased from 1,801 in 2019–2020 to 1,397 in 2021–2022, while emergency operations were stable (5,598 vs. 5,552), and total orthopedic ward staffing increased modestly (152 → 180 full-time equivalents). These factors may have influenced workflows. However, in sensitivity analyses of bays without PSM implementation, we did not observe a similar worsening of outcomes across the same time periods. Since staffing and surgical activity would be expected to affect all bays equally, it seems unlikely that these temporal changes alone explain the observed patterns. Instead, it is possible that initial reliance on the digital tool altered clinical focus and contributed to the observed changes, although we did not collect staff engagement data to confirm this. The following has been added to address this in the results and discussion:

Results:

“*Compared with the pre-implementation period (2019–2020), elective surgical activity was 22% lower in the intervention period (2021–2022), while emergency surgical activity remained stable (1%), and ward-level orthopedic nursing staffing increased by 18%.*”

Discussion:

“*While baseline patient characteristics were similar between periods, admittance patterns were nevertheless influenced by the pandemic, with fewer elective admissions. Other contextual shifts, such as increased staffing during the intervention period, may also have played a role. However, as these changes would be expected to affect all bays equally, and no comparable worsening of outcomes was observed in sensitivity analyses of control bays, they are unlikely to explain the observed patterns.”*

5. I have one other question: why did you use length of stay as your main outcome for a safety intervention? Death, ICU admission might be more appropriate?

**Our response:** Length of stay (LOS) was chosen as the primary outcome because it is a sensitive, patient-centered, and widely used metric in health services research, reflecting both clinical course and healthcare efficiency. As the intervention was designed to support detection and management of safety issues at the ward level, we considered LOS more likely to capture a broad impact on patient trajectories than rare outcomes such as in-hospital death or ICU admission. These latter outcomes were included as secondary endpoints, but given their low frequency in the studied orthopedic population, they were underpowered to detect meaningful differences. In contrast, LOS provided a more pragmatic and adequately powered measure of whether the PSM had an effect on patient safety and workflow.

Reviewer #2:

• This study presents a well-conducted, pragmatic pseudo-randomized trial evaluating the impact of electronic Patient Safety Monitors (PSMs) in a real-world hospital setting. Despite finding no significant reduction in hospital length of stay or key patient safety outcomes, the research offers valuable insights into the practical challenges of implementing PSMs. This work contributes meaningfully to the evidence base and highlights the need for further research into the effective integration of PSMs. Overall, this is a well-executed and timely study that offers valuable real-world insights into the limited effectiveness of digital patient safety interventions in routine clinical practice.

**Our response:** We thank the reviewer for the very positive evaluation of our study and for highlighting its contribution to the evidence base on digital patient safety interventions. We appreciate the recognition of the study’s real-world design and relevance.

• The manuscript would benefit from a brief justification for selecting orthopaedic bays as the setting for PSM implementation, as this could help readers assess the generalizability and contextual applicability of the findings. I am thinking several other clinical settings could be suitable—perhaps even more suitable—for evaluating the effectiveness of PMSs, especially if the goal is to assess broad patient safety outcomes, clinical workflow improvements, or preventable harms.

**Our response:** We agree that a brief justification for the selection of orthopedic bays for the setting for PSM implementation is warranted. We have now clarified our rationale for including orthopedic bays as the initial setting for PSM implementation. As described in the revised Methods section, orthopedic patients represent a relevant population for such interventions due to their high risk of preventable adverse events (e.g., pressure ulcers, venous thromboembolism, delirium, and falls). We believe this addition strengthens the contextual understanding and the generalizability considerations of our study.

Methods (Patient population and PSM implementation in the orthopedic ward):

*“Orthopedic patients were considered a relevant population for the initial implementation of PSMs, as this group is at increased risk of preventable adverse events such as pressure ulcers, venous thromboembolism, delirium, and falls, making patient safety monitoring particularly pertinent in this setting.”*

• The difference in LOS is statistically significant (p=0.046) but clinically trivial. The abstract and key messages say “no effect” on LOS, which might appear contradictory unless clarified as not clinically meaningful. The discussion restates that LOS increased slightly and was statistically significant, but does not highlight how small the actual difference was.

**Our response:** We thank the reviewer for highlighting this inaccuracy in our manuscript. We have revised the abstract conclusion and first paragraph of the Discussion to clarify that although LOS increased and the difference was statistically significant, the absolute difference was too small to be clinically relevant. We also note that the reviewer refers to the “Key Messages” section. Following editorial feedback and journal style requirements, we have removed this section in the revised manuscript.

Abstract Conclusion:
*“The implementation of PSMs in this study did not improve clinical or patient safety outcomes. Hospital length of stay was statistically longer after PSM implementation, although the difference was not clinically relevant.”*

Discussion:
*“The principal finding of our study was that implementation of electronic PSMs was associated with a statistically significant but clinically negligible increase in hospital LOS.”*

• They speculate about implementation challenges (training, workflow disruption), but no data on user engagement, usage logs, or staff feedback is presented or acknowledged as a gap.

**Our response:** We are grateful for this valuable comment, which we agree should be addressed in the limitations. As suggested, we have clarified this in the Strengths and Limitations section by explicitly noting that our study lacks both qualitative assessments (e.g., staff perceptions, usability evaluations) and quantitative measures (e.g., usage logs or user engagement metrics). We believe this addition highlights more clearly why the underlying causes for the limited effect of PSM could not be further explored.

Discussion (Strengths and limitations):
*“The study lacks qualitative assessments, such as staff perceptions or usability evaluations, and quantitative measures such as usage logs or other indicators of user engagement, which could have provided insights into the cause for the apparent limited effect of the PSM implementation.”*

• The risk of temporal confounding, especially given that the study spans the COVID-19 pandemic period, which could have independently influenced hospital workflows, admissions, and outcomes. You can be 1-2 sentences describing these issues!!

**Our response:** This is an important issue. As outlined in our response to Reviewer #1 (comment #4), we have already added contextual data to the Results and Discussion sections describing potential temporal confounding related to the COVID-19 pandemic, including reduced elective surgical activity, stable emergency operations, and increased staffing levels during the intervention period. In the Discussion, we also explicitly acknowledge that admittance patterns were influenced by the pandemic, but emphasize that these factors would be expected to affect both intervention and control bays equally, and therefore are unlikely to fully explain the observed results.

• The discussion is well-structured and places the study’s findings within the broader context of existing literature and global patient safety initiatives. The limitations are clearly presented and helpfully explain potential sources of bias, reflecting strong awareness of the study’s methodological constraints and enhancing its overall credibility. However, findings may not be generalizable to other healthcare settings with different organizational structures, patient populations, or digital infrastructure. Please considered to be as one of the limitations.

**Our response:** We agree that generalizability is a relevant concern. The Strengths and Limitations section has therefore been expanded to clarify that the findings may not be generalizable to other healthcare systems with different organizational structures, patient populations, or digital infrastructure.

Discussion (Strengths and limitations):

*“The study was conducted in the orthopedic bays of a single hospital, which limits the generalizability of the findings to other departments, hospitals, or healthcare systems, particularly those with different organizational structures, patient populations, or digital infrastructure.”*

• Although important variables such as age, sex, Charlson Comorbidity Index, and cause of admission were adjusted for, other potentially relevant confounders—such as staffing levels, nurse-to-patient ratios, concurrent safety initiatives, or recent policy changes—may not have been fully accounted for. Why?

**Our response:** We recognize that additional confounders may have played a role. As described in our response to Reviewer #1 (comment #4), we collected contextual data on staffing levels and surgical activity during the study period and have included these in the Results and Discussion. While these factors may have influenced workflows, they were similar across intervention and control bays and are therefore unlikely to fully explain the observed results. We acknowledge, however, that other unmeasured confounders—such as concurrent safety initiatives or broader policy changes—could not be fully accounted for and may have introduced residual confounding. This limitation has now been emphasized more explicitly in the Strengths and Limitations section.

Discussion (Strengths and limitations):

*“* *In particular, concurrent safety initiatives or policy changes at the hospital level could not be fully accounted for.”*

• Staff rotating between intervention and control bays or informal diffusion of PSM practices to non-PSM areas could dilute the observed effects, but this possibility is not well addressed.

**Our response:** This is a thoughtful observation. The main comparison in our study was between two time periods (2019–2020 vs. 2021–2022) in the same orthopedic bays before and after PSM implementation, so staff rotation between concurrent intervention and control units is not applicable. However, we also included two additional orthopedic bays without PSM implementation as a sensitivity analysis to account for temporal trends. While informal diffusion of PSM-related routines to these bays during the later time period cannot be fully excluded, we did not observe a significant change in LOS in these control bays, suggesting that such diffusion is unlikely to explain the results observed in the intervention bays. We now explicitly acknowledge this possibility in the Strengths and Limitations section.

Discussion (Strengths and limitations):

*“* *While informal diffusion of PSM-related practices to bays without PSM implementation during the later time period cannot be fully excluded, sensitivity analyses did not show significant changes in LOS in these control bays, suggesting that diffusion is unlikely to explain the findings.”*

6. PLOS authors have the option to publish the peer review history of their article ([what does this mean?](https://track.editorialmanager.com/CL0/https:%2F%2Fjournals.plos.org%2Fplosone%2Fs%2Feditorial-and-peer-review-process%23loc-peer-review-history/1/010f01982c534322-c1165aa2-4b1f-45af-b866-a28d73621516-000000/7V8UjZUb8qNWiEjd1qbjmnfiBYLUwr5dyZDS2km9NDg=220)). If published, this will include your full peer review and any attached files.

If you choose “no”, your identity will remain anonymous but your review may still be made public.

**Do you want your identity to be public for this peer review?** For information about this choice, including consent withdrawal, please see our [Privacy Policy](https://track.editorialmanager.com/CL0/https:%2F%2Fwww.plos.org%2Fprivacy-policy/1/010f01982c534322-c1165aa2-4b1f-45af-b866-a28d73621516-000000/UlF6C4TIHK_-lW6b-4unQUVLlQ_L7OfQmCwv0X4I-qM=220).

Reviewer #1: **Yes:** Christian P Subbe

Reviewer #2: No
